# Rosmarinic Acid as Bioactive Compound: Molecular and Physiological Aspects of Biosynthesis with Future Perspectives

**DOI:** 10.3390/cells14110850

**Published:** 2025-06-05

**Authors:** Dragana Jakovljević, Marzena Warchoł, Edyta Skrzypek

**Affiliations:** 1 Department of Biology and Ecology, Faculty of Science, University of Kragujevac, Radoja Domanovića 12, 34-000 Kragujevac, Serbia; dragana.jakovljevic@pmf.kg.ac.rs; 2 The Franciszek Górski Institute of Plant Physiology, Polish Academy of Sciences, Niezapominajek 21, 30-239 Krakow, Poland; m.warchol@ifr-pan.edu.pl

**Keywords:** rosmarinic acid, biosynthetic pathway, molecular mechanism, elicitation, metabolic engineering

## Abstract

The ester of caffeic acid with α-hydroxydihydrocaffeic acid, named rosmarinic acid (α-o-caffeoyl-3,4-dihydroxyphenyllactic acid; RA) can occur as oligomeric molecules, or in free, esterified, and glycosidic forms. Although it is commonly found among the members of the plants from the Lamiaceae (mints) and Boraginaceae (borages) families, only certain plant species produce a comparatively high concentration of RA. This valuable bioactive compound exhibits anti-cancer, anti-angiogenic, antioxidant, anti-inflammatory, antiviral, and antimicrobial properties, among others. As it is difficult to obtain high quantities of RA from natural sources, and since chemical manufacturing is costly and challenging, various biotechnological methods have recently been investigated to boost RA production. Plant cell tissue culture has been used to promote RA production in various plant species, particularly medicinal ones, with elicitation being the most commonly used technique. This review explores the main steps involved in RA biosynthesis in plants, including the molecular mechanisms and physiological alterations underlying its function, along with the primary mechanisms of RA accumulation in response to elicitation. Recent progress in synthetic biology-based RA synthesis, as well as metabolic engineering techniques to enhance the industrial production of this valuable bioactive constituent, are also discussed.

## 1. Introduction

Rosmarinic acid (α-o-caffeoyl-3,4-dihydroxyphenyllactic acid; RA) is an ester of caffeic acid with α-hydroxydihydrocaffeic acid. Its structure was reported in 1958 by Scarpati and Oriente [1], who were the first to isolate a previously undescribed main component of rosemary (*Salvia rosmarinus* Spenn.) leaves. Present-day studies have shown the occurrence of free RA in over a hundred plant species, ranging from 0.01 mg to 9.8 mg in 1 g of crude material, mainly in the Lamiaceae (mint) and Boraginaceae (borage) families, for which RA can be used as a chemotaxonomic determinant [2,3,4,5].

In the plant world, RA can occur in free, esterified, glycosidic forms, as well as in the form of oligomeric compounds. Unbound RA, its methyl ester, and melitric acids A and B have been isolated from the leaves of *Melissa officinalis* L. [6]. Melitric acids have a tridepside structure and are composed of three phenylpropanoic units. In relation to RA, they have an additional caffeic acid molecule attached by an ether bond. The further derivative of RA, sagerenic acid, present in *Salvia officinalis* L., is a dimer formed in the process of phytochemical cyclization [7]. Another compound, salvianolic acid K, was detected in the roots of *Salvia deserta* Schangin. and in *Salvia officinalis* L. [7]. The glycosidic form of RA is extremely rare; only a few compounds of this type have been described so far, mainly involving glucose [8]. These include 4′-O-β-d-glucopyranoside and 4,4′-O-di-β-d-glucopyranoside of RA, isolated from the fruits of the Indonesian medicinal plant *Helicteres isora* L. Both components show properties analogous to their aglycone, resulting from the presence of caffeoyl residues in the structure. Still, the main structural variation in RA occurs when two RA units combine to produce a compound, such as salvianolic acid B. Numerous related salvianolic acid derivatives and lithospermic acids seem to have their origins in this molecule as a precursor [9].

The free form of RA has been found in the following taxa: Lamiaceae (*Agastache*, *Coleus*, *Dracocephalum*, *Esholtzia*, *Glechoma*, *Hyptis*, *Hyssopus*, *Lavandula*, *Lycopus*, *Majorana*, *Melissa*, *Mentha*, *Monarda*, *Nepeta*, *Ocimum*, *Origanum*, *Orthosiphon*, *Perilla*, *Prunella*, *Rabdosia*, *Rosmarinus*, *Salvia*, *Satureja*, and *Thymus)*; Apiaceae (*Sanicula europaea* L.); Boraginaceae (*Anchusa*, *Lithospermum*, and *Symphytum)*; Plantaginaceae (*Plantago lagopus* L.); Sterculiaceae *(Helicteres isora* L.); and Sterculiaceae (*Helicteres isora* L.) [2]. Among the above-mentioned taxonomic groups, only a few plants have a relatively high concentration of RA. Due to the wide range of pharmacological properties they are commonly used in medicine to treat and prevent many diseases.

RA demonstrates anti-cancer, anti-angiogenic, antioxidant, anti-inflammatory, anti-viral, and anti-microbial properties by preventing cell damage caused by free radicals [4,5,10]. As a naturally occurring substance, RA has high therapeutic potential, as confirmed by both in vitro and in vivo studies. It stabilizes biological membranes, protects against the harmful effects of UV radiation and reactive oxygen species, and exhibits sedative effects on the central nervous system. It also has minor anti-allergic properties, and its ester derivatives possess choleretic properties. RA displays characteristics of a non-specific inhibitor of enzymes, reducing the activity of cyclooxygenase 1 and 2, lipoxygenase, hyaluronidase, b-hexosaminidase, ornithine decarboxylase, aldose reductase, α-amylase, carboxypeptidase A, adenylyl cyclase, and iodothyronine deiodinase to varying degrees. It stimulates the production of prostaglandin E2 and reduces the synthesis of leukotrienes B4 in leukocytes. It also has a protective effect on plasma lipoproteins, including the LDL fraction. Together with rutin, ursolic acid, or lycopene, it shows synergistic properties. RA reduces the permeability of capillary vessels, preventing the penetration of toxins and microorganisms. It also decreases the synthesis of inflammatory mediators (prostaglandins, prostacyclins, and thromboxane), thereby eliminating their effect on sensory endings and preventing platelet aggregation. RA also shows antimicrobial properties. Its virostatic activity against herpes simplexvirus type 1 (HSV-1) and human immunodeficiency virus (HIV-1) is due to its interaction with the viral and host protein structures, which delays the adsorption on the cell surface or impairs viral replication. As a nonspecific enzymatic inhibitor, RA reduces in vitro the activity of key HIV replication enzymes, reverse transcriptase, and integrase, thereby preventing the synthesis of a complementary viral DNA strand on the RNA matrix and its association with the host chromosome [5,10,11,12].

RA also plays an important role in the growth promotion and protection mechanisms against biotic and abiotic stresses in plants [13,14,15,16,17]. It shows strong antioxidant properties, which are essential for scavenging free radicals and defending cells from oxidative stress. The secretion of RA was observed in the roots of *Ocimum basilicum* L. when it was infected with the fungal pathogen *Pythium ultimum*, as well as during *Fusarium* attacks in barley [18,19]. RA can also be used for food preservation due to its ability to inhibit lipid peroxidation and the growth of bacteria, as well as an antibiotic substitute [20,21]. It is further applied in cosmetic preparations due to its antioxidant and anti-inflammatory properties [20].

In the natural environment, plants produce small amounts of RA, and its chemical synthesis is difficult and expensive. In order to increase production, various biotechnology approaches are being investigated. RA production using plant cell tissue culture has been carried out in numerous plant species, especially medicinal ones. The optimization of media and culture conditions, along with elicitation, are commonly applied methods to improve the production of secondary metabolites in cell cultures. Most plants do not produce secondary metabolites in satisfactory quantities, and their amounts are often inconsistent [20]. RA is accumulated very intensively in undifferentiated cell suspension, callus cultures, or shoot cultures—often in much higher quantities compared with whole plants [3,13]. Besides cell and callus culture, hairy roots culture appears to be a promising method for RA production. The success of RA production using in vitro systems has enabled the industrial-scale synthesis of RA in bioreactors [22]. RA production has also been achieved by expressing its biosynthesis enzymes from plants in yeast (*Saccharomyces cerevisiae* Meyen ex E.C. Hansen). This pilot study confirmed the opportunity to develop a fermentative procedure for RA production using *Saccharomyces cerevisiae* cells [23].

The interest in RA has continuously increased since it was first described. Recent research has explored not only the biosynthesis pathway and factors influencing the accumulation of RA in plant tissues but also the transcriptome level and genes encoding biosynthesis. The objective of this review is to present the molecular and physiological aspects of RA biosynthesis with future perspectives—especially the optimization of RA production methods for commercial use.

## 2. Rosmarinic Acid Biosynthetic Pathways in Plants

There are not many enzymes unique to the biosynthetic pathway required for RA synthesis. It is now believed that a limited number of enzymes needed to be “invented” specifically for the biosynthesis of RA, most likely based on genes involved in the production of caffeoylshikimic acid and chlorogenic acid. Additional biosynthetic steps may have been drawn from photorespiration, tocopherol/plastoquinone biosynthesis, and phenylpropanoid metabolism [24].

**Figure 1:**
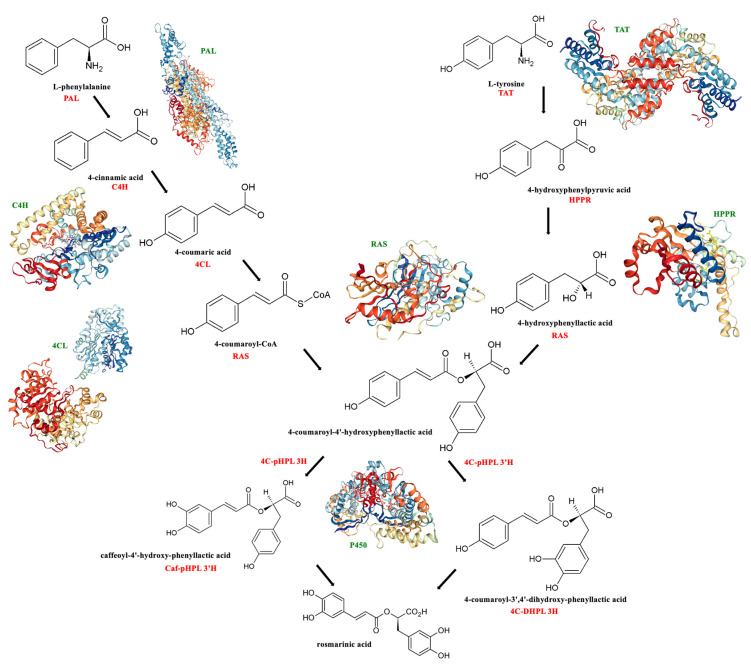
Commonly proposed biosynthetic pathway of rosmarinic acid (redrawn from Petersen et al., 2009 [24]) with structure of enzymes involved. PAL—phenylalanine ammonia lyase; C4H—cinnamic acid 4-hydroxylase; 4CL—4-coumaric acid CoA-ligase; TAT—tyrosine aminotransferase; HPPR—hydroxyphenylpyruvate reductase; RAS—hydroxycinnamoyl-CoA:hydroxyphenyllactate hydroxycinnamoyltransferase (rosmarinic acid synthase); 4C-pHPL 3H, 4C-pHPL 3′H—4-coumaroyl-4′-hydroxyphenyllactate 3/3′-hydroxylases; Caf-pHPL 3′H—caffeoyl-4′-hydroxyphenyllactate 3′-hydroxylase; 4C-DHPL 3H—4-coumaroyl-3′,4′-dihydroxyphenyllactate 3-hydroxylase. The abbreviations of the enzyme names in the rosmarinic acid biosynthetic pathway are marked in red, and those of the structural formulas are marked in green. The structures of the presented enzymes are downloaded from www.brenda-enzymes.org (accessed on 12 March 2025).

Beginning with the aromatic amino acids L-phenylalanine and L-tyrosine, the biosynthesis continues with their independent transformation [25]. The biosynthetic pathways starting with both amino acids, along with the enzymes involved in RA synthesis, are presented in Figure 1.

### 2.1. L-Phenylalanine

The enzyme phenylalanine ammonia-lyase (**PAL**; E.C. 4.3.1.5) catalyzes the initial step in the metabolism of phenylpropanoid compounds—a reaction involving the deamination of phenylalanine to cinnamic acid [26]. Through this common step, numerous aromatic metabolites are produced. They can be divided into classes (or subclasses) that include lignins, flavonoids, phenolic acids, coumarins, and stilbenes, among others. Variations in PAL enzyme activity can be attributed to abiotic and biotic environmental conditions [13]. In addition to the 4-methyldiene-imidazol-5-one (MIO) co-factor in the active site, this helix-containing protein has two structural segments—the N-terminal mobile extension and the shielding domain over the active center [27]. The expression of the PAL activity genes is spatially and temporally controlled. It is known that the synthesis of secondary metabolites cannot be regulated until adequate primary metabolite synthesis and photosynthetic activities have occurred. Although the PAL enzyme cannot be considered a component of the plant antioxidant system, the compounds it produces contribute significantly to the plant’s overall antioxidant capacity [28].

Following the deamination of L-phenylalanine to cinnamic acid, the cytochrome P450 monooxygenase cinnamate 4-hydroxylase (**C4H**; E.C. 1.14.13.11) then hydroxylates the fourth carbonic atom of the benzene ring in the cinnamic acid, forming 4-coumaric acid [22,29]. Hydroxycinnamic acids must be activated before proceeding to the next step. These acids come in a variety of activated forms. Although the coenzyme A thioester form is the most prevalent, hydroxycinnamic acid can also be activated via glucose esters or cinnamic acid esters (such as chlorogenic). In addition, 4-coumaroyl-coenzyme A ligase (**4CL**; E.C. 6.2.1.12) participates in the activation process [3], resulting in the formation of the intermediatory precursor 4-coumaroyl-CoA [22].

### 2.2. L-Tyrosine

Tyrosine is produced de novo in plants through the shikimate pathway, which also produces tryptophan and phenylalanine, the other two aromatic amino acids [30,31]. Tyrosine is a biosynthetic precursor to ubiquinone, plastoquinone, and tocopherols, all of which are necessary for plants to function. Additionally, RA and its derivatives in the Lamiaceae and Boraginaceae families are among the many specialized metabolites that are synthesized by several plant groups using tyrosine as a substrate [3]. The reversible transamination from tyrosine to 4-hydroxyphenylpyruvic acid (**pHPP**)—the first stage of the tyrosine conversion—is catalyzed by tyrosine aminotransferase (**TAT**; EC 2.6.1.5). Aromatic aminotransferases are typically located in the cytosol of plants. The importance of TAT as an entry-point enzyme in tyrosine-derived RA biosynthesis has been extensively studied [32,33,34,35,36], even though they are less significant in the biosynthesis of aromatic amino acids [37]. In order to function, aminotransferases need pyridoxal-5-phosphate. In *Anchusa officinalis* cell cultures, TAT demonstrated substrate selectivity to tyrosine, as well as to other amino moiety acceptors such as oxaloacetate and α-ketoglutarate [22].

In general, aminotransferases can be divided into four subgroups. According to Mehta et al. [38], aromatic aminotransferases and aminotransferases that transaminate aspartate or alanine are found in subgroup I. One large and one small domain are the main parts of the homodimeric aminotransferase’s two subunits. The contact between the subunit and the two domains forms the catalytic center. Pyridoxal-5′-phosphate (PLP) needs a conserved Lys-residue whose ɛ-amino group forms an internal aldimine with PLP in order to be covalently linked to the enzyme’s active site. The external aldimine is formed when the reacting amino acid displaces the existing group. After the α-proton is separated and hydrolyzed, the 2-oxoacid is eventually produced. The accepting 2-oxoacid—which is then transformed into the matching amino acid—regenerates the pyridoxamine-5′-phosphate, which is still attached to the enzyme, in the second reaction phase. Therefore, the entire process is categorized as a ping-pong mechanism from a mechanistic standpoint [37,39].

In a further step, the 4-hydroxyphenyllactic acid (pHPL) is produced via the reduction of pHPP by hydroxyphenylpyruvate reductase (**HPPR**, EC 1.1.1.237). In this reaction, both NADH and NADPH can serve as co-substrates. Although it has a modest affinity, HPPR also lowers the concentration of 3,4-dihydroxyphenylpyruvate [22,31,40].

### 2.3. Rosmarinic Acid Synthase

4-hydroxyphenyllactate, derived from tyrosine, and 4-coumaric-CoA, derived from phenylalanine, are coupled by an ester bond in the subsequent biosynthesis phase. The enzyme 4-coumaroyl-CoA:4′-hydroxyphenyllactic acid 4-coumaroyltransferase, also known as rosmarinic acid synthase (**RAS**; E.C.2.3.1.140), catalyzes the reaction. Through the activity of RAS, 4-coumaroyl-4′-hydroxyphenyllactic acid (4C-pHPL) is created by the formation of an ester bond between two intermediate precursors, with the concurrent release of coenzyme A. In this step, the carboxyl group of 4-coumaric acid and the hydroxyl group of 4-hydroxyphenyllactate create an ester bond. This ester—known as 4-coumaroyl-4-hydroxyphenyllactate—is further hydroxylated on the 3 and 3′ locations of the aromatic ring, where cytochrome P450-dependent monooxygenase processes are primarily responsible for the introduction of the 3- and 3′-hydroxyl groups. [22,29]. According to Matsuno et al. [41], three cytochrome P450s are involved in RA biosynthesis, including C4H (identified as CYP73A3 and CYP73A30), and CYP98A6, which catalyzed the 3-hydroxylation of the hydroxycinnamoyl moiety of R-CHPL to form cafeoyl-4′-hydroxyphenyllactic acid, an immediate precursor of RA. Even though the precise biosynthetic mechanism of this conversion has not yet been fully characterized, RA may be further transformed into salvianolic acid B and lithospermic acid B [42,43].

**Figure 2:**
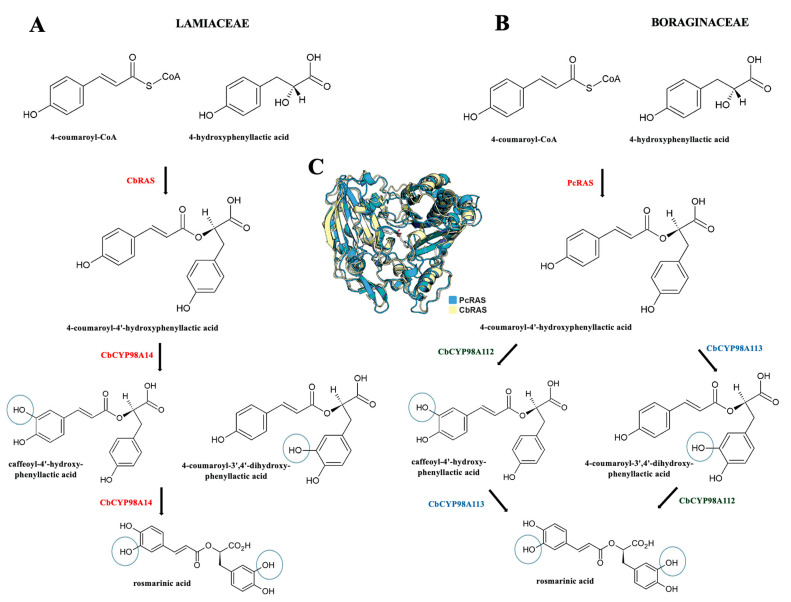
Comparison of (**A**) Lamiaceae and (**B**) Boraginaceae biosynthetic pathways to rosmarinic acid starting from 4-coumaroyl-CoA and 4-hydroxyphenyllactic acid and an order of meta-hydroxylation of 4-coumaroyl-4′-hydroxyphenyllactic acid based on different activities of P450 hydroxylases from CYP98A family; (**C**) the structure of *Coleus blumei* RAS (CbRAS) and a structural model of *Phacelia campanularia* RAS (PcRAS) models (proposed by Levsh et al. [47]).

Among the enzymes involved in RA biosynthesis, RAS can be regarded as the most specific [22]. The enzyme BAHD acetyltransferase has a molecular mass of 47,932 Da. The RAS amino acid sequence was elucidated from *Coleus blumei* [44], following full-length cDNA isolation (1290 bp long coding for 430 amino acids) and expression in *E. coli.* The RAS cDNA was also isolated from *Melissa officinalis* [45] and *Lavandula angustifolia* [46]. Recently, Levch et al. [47] performed a molecular phylogenic analysis involving a variety of Lamiaceae and Boraginaceae species, where a maximum likelihood phylogeny of BAHD acetyltransferase closely related to *Phacelia campanularia* RAS (PcRAS) revealed a Boraginaceae clade distinct from the Lamiaceae RAS clade. Figure 2 summarizes the independent biosynthesis of RA in both the Lamiaceae and Boraginaceae families based on the RAS assays.

As mentioned previously, the occurrence of RA is mostly associated with the Boraginaceae and Lamiaceae families. In this context, the cDNAs encoding RAS, along with a cytochrome P450 enzyme (CYP98A14) responsible for catalyzing the 3- and 3′-hydroxylation, have been cloned and heterologously expressed in the established RA biosynthetic pathway from *Coleus blumei* (Lamiaceae) [44,48]. More precisely, it has been discovered that CYP98A14 catalyzes a single meta-hydroxylation reaction on each of the aromatic rings of 4-coumaroyl-(R)-3-(4-hydroxyphenyl)lactate to produce RA, following RAS catalyzed-transfer of the 4-coumaroyl acyl group from 4-coumaroyl-CoA to the acyl acceptor molecule. Furthermore, the main differences among the above-mentioned plant families regarding RAS activity and RA biosynthesis are outlined in the study by Levsh et al. [47]. These authors combined a multi-omics approach with both the in vivo and in vitro functional characterization of candidate genes and discovered that two cytochrome P450 hydroxylases (PcCYP98A112 and PcCYP98A113) possessed specific activities during RA production in *Phacelia campanularia* (Boraginaceae). Namely, the findings indicates that, when it came to the P450 activities, the PcCYP98A112 catalyzed the 3-hydroxylation of the acyl–donor phenolic ring, while the acyl–acceptor ring was catalyzed by PcCYP98A113. It should be noticed that, although the precise catalytic order of the 4-coumaroyl-(R)3-(4-hydroxyphenyl)lactate remains unclear from the in vitro study, the in vivo results indicate that the in vivo RA production involves the combination of both P450 hydroxylases. Additionally, Zhou et al. [49] pointed to the distinct evolution of various RASs; RASs from the Lamiaceae family originate from early evolved shikimate/quinate hydroxycinnamoyl transferases (HCTs), while a spermidine hydroxycinnamoyl transferase (SHT) ancestor are related to the Boraginaceae RASs.

## 3. Molecular Mechanisms of Rosmarinic Acid Accumulation

Plant-produced chemicals can be separated into two categories—primary and secondary metabolites. Primary metabolites are essential for growth and development, while secondary metabolites provide a complex way for plants to adapt to unfavorable environmental conditions [50,51]. The chemical structure, position, type, quantity, and diversity of functional groups define their well-established antioxidant action [52]. PAL, as a key enzyme involved in phenylpropanoid metabolism, is sensitive to environmental stimuli. Consequently, plants may react to stress by changing the activity of PAL and accumulating phenylpropanoids. As a result, the ratio of various secondary metabolites varies greatly within and between taxa, as well as depending on the habitat, season, and plant material. Additionally, an increased amount of bioactive compounds may be induced in stressed plants compared to non-stressed plants. This can result in distinct plant scents, tastes, and quality, as well as altered yield potential [53,54]. For example, since the glycolysis process and amino acid metabolism (including amino acids from the alanine group) may be enhanced under the conditions of nutrient limitation, it is shown that PAL activity and other early stages of the phenylpropanoid pathway can be stimulated. This may lead to an increased synthesis of valuable secondary metabolites, including RA [13,55,56]. By studying the expression profiling of RA biosynthetic genes and some physiological responses from *Mentha piperita* L. under salinity and heat stress, Gholamnia et al. [57] demonstrated a significant decrease in *RAS* expression and a significant increase in *C4H* and *HPPR* expression in plants subjected to simultaneous stresses. In the same study, salinity at 25 °C raised the relative levels of proline and phenolic compounds, while after 72 h at a salinity of 120 mM at 35 °C, rosmarinic acid, soluble sugar, chlorophyll, and the K^+^/N^+^ ratio all declined by factors of 3.2, 1.8, 4.6, and 9, respectively.

The production of secondary metabolites can increase or decrease by up to 50% as a result of environmental influences [58]. Environmental effects on secondary metabolite biosynthesis have been explained by two proposed mechanisms—passive and active. The so-called “passive shift” involves using plant energy to produce active substances as defense compounds to protect plant survival, as seen in growth reduction [59], while the “active shift” involves upregulating the enzymes involved in secondary metabolism [60]. Plants use their receptors to recognize these harmful signals from the environment and initiate defense mechanisms, such as secondary metabolism, in response to stressful conditions [61]. For example, plants use receptors attached to the plasma membrane to identify pathogen-derived elicitors, which trigger the synthesis of low-molecular-weight chemicals (e.g., rosmarinic acid) as a defensive mechanism.

Elicitation is one of the best methods for improving the biotechnologically generated secondary metabolites. One of the primary techniques used to generate cell cultures with induced metabolite synthesis is the application of biotic or abiotic elicitors to the culture media [62]. From a biotechnological perspective, an elicitor can be regarded as an environmental element or a signal molecule that initiates a signal–transduction cascade that modulates the expression of genes linked to the production of secondary metabolites. An array of metabolic alterations is systemically started in plant cells to activate the plant’s innate immune system upon elicitation challenges [62,63]. Due to numerous interconnected reactions, the elicitation mechanism is highly complex. Furthermore, the elicitors’ origin, specificity, and concentration, as well as the plants’ development stage, the physiochemical environment of the contact, etc., all affect these occurrences [61]. Usually, plants’ response to elicitors starts in the cell’s plasma membrane. Although they may trigger the same signaling pathways, different membrane receptors perceive different elicitors [64]. Isolating elicitor signal molecules and identifying the associated receptors have been challenging, but it seems that different plant species may have common receptors for their recognition. For a variety of elicitors with different chemical structures, a number of elicitor-binding sites have been found in cell membranes. Although broad-spectrum elicitors can activate defenses in cultivars of several species, pathogen avirulence (Avr) genes and plant resistance (R) genes are essential in plant innate immunity, where specialized Avr gene products trigger defense responses only in cultivars with corresponding R genes [65]. The phytoalexinix activities of RA were demonstrated in the case of *Ocimum basilicum* roots. Namely, *O. basilicum* roots enhanced the RA production 2.67-fold upon elicitation with *Pythium ultimum*. Furthermore, RA showed antimicrobial activity against *Pseudomonas aeruginosa* and various soil-borne microorganisms. According to Bais et al. [18], RA is an essential antimicrobial component found in nature that, when challenged by microbes, may be released into the surrounding rhizosphere. Plant cells identify conserved microbe-associated molecular patterns (MAMPs)—a new term for universal and exogenous elicitors—to initiate defense responses, according to the novel explanation for plant innate immunity. On the other hand, a pathogen invasion may trigger the production of endogenous molecules or elicitors in plants, which are known as danger-associated molecular patterns. The identification of pathogen-secreted effectors, formerly referred to as particular elicitors, which are members of several groups such as proteins, glycans, and lipids, constitutes a second level of perception [62,66]. Today, it is well known that plant defense signaling molecules that mediate the plant defense response—such as methyl jasmonate (MeJA), salicylic acid (SA), jasmonic acid (JA), among others—can act as elicitors and cause the increased accumulation of RA. Table 1 summarizes the main elicitors commonly used to induce the accumulation of RA.

### Effect of Elicitor on Rosmarinic Acid Accumulation

The effects of the foliar application of MeJA (50 μM) and AgNO_3_ (15 μM) on RA accumulation and the expression of PAL, TAT, HPPR, RAS, and CYP98A14 genes in *Salvia officinalis* and *S. verticillata* were assessed by Pesaraklu et al. [67]. The treatment of MeJA and Ag^+^ significantly increased the accumulation of RA in both of the tested species, according to the results. Furthermore, MeJA and Ag^+^ influenced the expression of the key genes *PAL*, *TAT*, *HPPR*, *RAS*, and *CYP98A14* in both the phenylpropanoid and tyrosine pathways. Kianersi et al. [68] investigated the effects of MeJA on RA content and the alterations in the expression of key genes associated with biosynthesis (*MoPAL*, *Mo4CL*, and *MoRAS*) in Iranian lemon balm ecotypes (*Melissa officinalis* L.). According to the results, MeJA dosages considerably increased the RA content in both ecotypes when compared to the control samples. For both Iranian lemon balm ecotypes, RA accumulation was associated with increased expression levels of *MoPAL*, *Mo4CL*, and *MoRAS* in all treatments. In order to investigate whether MeJa is related to a signal transduction system in *Prunella vulgaris* hairy roots, Ru et al. [69] treated the hairy roots with MeJA and measured the accumulation of RA. The best induction efficiency—measured five days after application—was with 100 μM MeJA. Furthermore, a correlation study revealed that RA accumulation was substantially linked with *PvPAL*, *PvHPPR*, *PvC4H*, *Pv4CL1*, *Pv4CL2*, and *PvCYP98A101* transcript expression, but not with *PvRAS* and *PvTAT* transcripts. Kianersi et al. [70] investigated the molecular mechanism of RA accumulation in two *Salvia* species (*Salvia yangii* and *S. abrotanoides*) upon MeJA elicitation. They found that, in comparison with untreated plants, 150 M MeJA increased RA content in *S. yangii* and *S. abrotanoides* by 1.66- and 1.54-fold, respectively. The effects of MeJA are most likely due to the activation of genes involved in the phenylpropanoid pathway, as evidenced by the increased numbers of transcripts for RAS, 4CL, and PAL. According to the study of Xiao et al. [86], the production of RA can be elevated by genetic manipulation, since the hairy root culture of *Salvia miltiorrhiza* produced several times higher concentration of RA due to the overexpression of a single gene (*c4h*, *tat*, and *hppr*), the overexpression of both *tat* and *hppr*, as well as the suppression of *hppd*. In the study by Khojasteh et al. [71], 100 μM MeJA was tested on cell suspension cultures of *Satureja khuzistanica*. Without influencing biomass production, MeJA more than tripled RA productivity, with the stimulated cultures reaching 3.9 g L^−1^. A maximum RA production of 3.1 g L^−1^ and biomass productivity of 18.7 g L^−1^ per day under MeJA elicitation were obtained when the cell culture was moved from a shake flask to a wave-mixed bioreactor. The production of RA was significantly enhanced by the elicitation period and concentration of MeJA in the hairy root culture of *Lepechinia caulescens*. When the treatments were elicited for 24 h, the highest accumulation occurred at a concentration of 300 µM of MeJA [72]. In the experiment with cell cultures of *Agastache rugosa*, the transcript levels of *ArPAL*, *Ar4CL*, and *ArC4H* increased 4.5-fold, 3.4-fold, and 3.5-fold, respectively, compared with the untreated controls, and the culture contained relatively high amounts of RA after exposure of cells to 50 µM MeJA [73]. The results also indicate that both RA and precursors—such as shikimate and aromatic amino acids—were induced as a response to MeJA treatment. In cell suspension cultures of *Mentha* × *piperita*, the effects of JA and MeJA on RA accumulation and cell development were also studied [74]. A total of 24 h after adding 100 μM MeJA, the maximum RA accumulation (117.95 mg g^−1^ DW) was recorded. Then, 48 h following the application of 200 μM JA, a comparable quantity (110.12 mg g^−1^ DW) was found. These values were over 1.5 times higher than those of the elicitation-free control sample. Elicitors had no noticeable impact on the secretion of RA into the culture media. RA concentrations outside of cells were comparable to those found in the control group. When compared to the control, the suspension cultures of *M*. *piperita* treated with elicitors were shown to have lower biomass accumulation. Zhou et al. [75] analyzed a transcriptome library of *Salvia miltiorrhiza* in response to MeJA. In addition to upregulating the expression of genes encoding important enzymes in the RA biosynthesis pathway, such as CYP98A14, overexpressing *SmMYB1* markedly increased RA accumulation. *SmMYB1* was found to activate the expression of CYP98A14 and genes encoding anthocyanin biosynthesis pathway enzymes, such as chalcone isomerase (CHI) and anthocyanidin synthase (ANS), according to dual-luciferase (dual-LUC) assays and/or electrophoretic mobility shift assays (EMSAs). Furthermore, it was demonstrated that, in contrast to *SmMYB1* acting alone, the combination of *SmMYB1* and *SmMYC2* increased *CYP98A14* expression. The level of gene expression for proteins associated with the RA biosynthesis pathway in *Origanum vulgare* in a consortium of three bacteria (*Azotobacter chroococcum*, *Azospirillium brasilense*, and *Pseudomonas fluorescens*) at two salinity levels and two elicitor levels (0.1 and 0.5 mM MeJA) were studied by Jafari et al. [76]. The findings demonstrated that, among all treatments, salt had a beneficial impact on DPPH radical scavenging capacity and a negative impact on RA induction and RAS gene expression. When 0.1 mM MeJA and bacterial consortia were applied together without salt, the expression of the RAS and C4H genes increased 3.37 and 6.6 times, respectively, in comparison to the control.

The results obtained by Gonçalves et al. [87] suggest that shoots of *Thymus lotocephalus* are a good source of antioxidant compounds, and that RA accumulation can be promoted through SA application and alterations in vitro culture conditions. In the callus culture of *Salvia nemorosa*, 0.5 μM SA increased RA content eight times compared to the control [77]. Li et al. [78] investigated the synthesis of RA under SA-induced pH changes in *Salvia miltiorrhiza* suspension cells, along with the *PAL*, *TAT*, and *RAS* gene expression. It was shown that SA decreased the cytosolic pH by inhibiting the activity of plasma membrane H^+^-ATPase. SA also upregulated the gene expression of *TAT*, *PAL*, and *RAS*, and as a result, enhanced the accumulation of those phenolic acids. Guo et al. [88] found that SA application led to the cytoplasmic acidification of *Salvia miltiorrhiza* cells and the alkalinization of the extracellular medium. Furthermore, Ca^2+^ protoplast mobilization was induced by SA, but the induction could be blocked by either organellar or plasma membranes, or a channel blocker. In the same study, both PAL activity and its gene expression showed the same patterns. On the other hand, a single application of salicylic acid (10 μM) on *Thymus membranaceus* culture media resulted in an increase in RA and phenolic levels, which in turn improved the extracts’ antioxidant properties [79]. The exogenous application of 250 and 500 μM SA led to the upregulation of *PAL* expression in *Salvia officinalis* (*SoPAL*) and *S. virgata* (*SvPAL*) [80]. Further analysis showed that, in *S. virgata*, at 500 μM SA, a higher accumulation of RA was achieved, while in *Salvia officinalis*, a higher RA accumulation was observed at 250 μM SA. It was determined that the RA accumulation in the tested species did not positively correlate with the level of *PAL* transcription. Thus, lower levels of RA were accumulated in the case of *S. officinalis*, even if the transcription rate of *PAL* increased at greater concentrations of SA. The synergistic effects of SA and H_2_O_2_ on RA production in *Salvia miltiorrhiza* cell cultures were investigated by Hao et al. [81]. The findings demonstrated that SA markedly increased RA accumulation, PAL activity, and H_2_O_2_ generation. Additionally, exogenous H_2_O_2_ increased RA synthesis and PAL activity. However, RA formation could be prevented if a quencher (DMTU) or an NADPH oxidase inhibitor (IMD) were introduced to suppress H_2_O_2_ generation. According to these findings, H_2_O_2_ is a secondary messenger for signal transduction, which SA can strongly activate, and which encourages the production of RA. The study of Khalil et al. [82] aimed to investigate the effect of applying various concentrations of SA on *Thymus vulgaris* L., while subjecting the plant to decreasing amounts of irrigation water. RA was detected as the major component with a higher percentage observed upon subjecting the plant to 3 mM SA. Stasińska-Jakubas et al. [83] foliar-applied three different elicitors (150 mg/L chitosan lactate (ChL), 10 mg L^−1^ selenite (Se), and 100 mg L^−1^ SA) to estimate the effect on the primary and secondary metabolism of *Melissa officinalis*. The applications of ChL and SA had the strongest elicitation impact, while the application of Se produced a slightly weaker effect. Furthermore, the highest concentration of RA was noted on the eighth day after elicitation, independent of the type of elicitor. Elicitation had the strongest effect on rosmarinic acid hexoside among the metabolites examined, with its level increasing several-fold compared to the control. Although some elicitor-induced alterations in parameters linked to photosynthesis and oxidative stress were observed, no apparent signs of chemical toxicity or impact on plant biomass were discovered.

In the study by Park et al. [84], the treatment of *Agastache rugosa* cell cultures with 500 mg L^−1^ YE and 30 mg L^−1^ silver nitrate increased the synthesis of RA and the expression of genes involved in the phenylpropanoid pathway. The quantity of YE and silver nitrate was correlated with the expression of *RAS* and *HPPR*. While *PAL* expression with silver nitrate treatment was 52.31 times higher than in the untreated controls after 24 h of elicitation, the transcript levels of *HPPR* under yeast extract treatment were 1.84-, 1.97-, and 2.86-fold higher than the control treatments after 3, 6, and 12 h, respectively. Additionally, YE supplementation recorded the highest quantity of RA at 4.98 mg g^−1^. The effects of YE on *TAT* gene expression and RA accumulation in *Melissa officinalis* seedlings were examined by Nasiri-Bezenjani et al. [85]. For the 17 h treatment, YE doses of 0.05%, 0.1%, and 0.2% markedly stimulated the RA biosynthesis. At 0.1% YE treatment, when RA biosynthesis significantly increased, the highest *TAT* gene expression was observed. The authors suggested that these findings could be linked to the oxidative stress that YE causes, as seen from the rising catalase and superoxide dismutase activity.

A considerable amount of research is focusing on the elucidation of RA biosynthesis and accumulation. Although the pathways of transduction and responsive genes have been clarified in some cases, it should be taken into account that different elicitors may be perceived by distinct membrane receptors, and that there is a broad variety of responses due to heterogeneity in the way of action. The general mechanism of action of elicitors was investigated by Ramirez-Estrada et al. [62]. In brief, second messengers are involved in the signal transduction pathways triggered by elicitors, which are first recognized by specific receptors. These messengers amplify the signal, initiating additional downstream reactions. The following is a summary of the sequential steps that occur in elicitor-induced defense reactions: perception of elicitor by receptor; reversible phosphorylation and dephosphorylation of proteins from plasma membrane and cytosol; cytosolic (Ca^2+^) enhancement; Cl^−^ and K^+^ efflux/H^+^ influx (cytoplasmic acidification and extracellular alkalinization); activation of MAPK; activation of NADPH oxidase and reactive oxygen and nitrogen species (ROS and RNS) production; expression of early defense genes; production of jasmonate; expression of the late defense response gene; and accumulation of secondary metabolites. According to Shakya et al. [61], ROS generation is an important aspect, since there is a linked role of G-proteins, ion channels, protein kinases, MAPKs, gene expression, and enzymatic reaction, which may reprogram the pathway of secondary metabolite production. All of the abovementioned should be taken into account when considering elicitor application for the induced synthesis of RA.

## 4. Synthetic Biology and Rosmarinic Acid Synthesis

The growing need for plant polyphenolic compounds cannot be met only by extracting phenolic compounds from natural plants, especially when it comes to RA, as previously discussed. As an alternate method for obtaining plant bioactive phenolic compounds, heterologous synthesis in plants has become popular in recent years. The benefit of this strategy is that since the phenylpropanoid pathway is already established, fewer genes need to be added. Plants, however, have competing pathways that have the potential to significantly disrupt the synthesis of polyphenols. Furthermore, the growth of these heterologous plants is influenced by the availability of fertile land and suitable weather conditions; this is a time-consuming process, as these plants are very challenging to genetically modify. Additionally, using plant cell cultures can have certain drawbacks, including heterogeneity, inconsistent yields, slow growth rates, unstable cultures, and vulnerability to aggregation and stress [89,90].

One of the most efficient ways to overcome this restriction and to generate large quantities of beneficial compounds, including RA, may be to create microbial cell factories that can produce polyphenols effectively. The development of synthetic biology techniques and the discovery of novel isoenzymes in plants or less complex organisms to construct heterologous pathways have made it easier to create effective microbial cell factories. Attempts have been made to enhance the host chassis in order to increase the process’ profitability [91]. These methods offer consistent quality, scalability, accessible extraction techniques, and the possibility of increased synthesis efficiency because they use renewable lignocellulosic biomass as a carbon source, which lessens the requirement for hazardous chemicals. Furthermore, the chemical variety of natural products, whose structural complexity might occasionally be difficult to obtain by multistep chemical synthesis, could be increased through biological synthesis [90].

**Figure 3:**
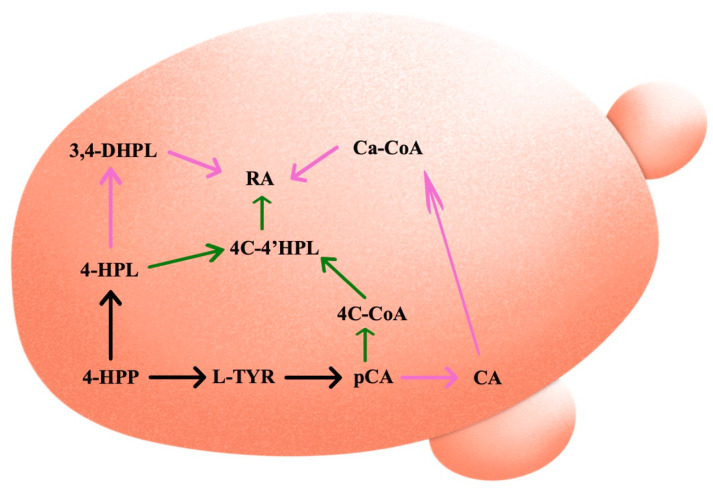
Simplified route of rosmarinic acid (RA) synthesis in *Saccharomices cerevisiae*. 4-HPP, 4-hydroxyphenylpyruvate; 4-HPL, 4-hydroxyphenyllactate; 3,4-DHPL, 3,4-dihydroxyphenyllactate; L-TYR, L-tyrosine; pCA, p-coumaric acid; CA, caffeic acid; 4C-CoA, 4-coumaroyl-coA; 4C-4′HPL, 4-coumaroyl-4′-hydroxyphenyllactic acid; Ca-CoA, caffeoyl-coA; RA, rosmarinic acid. The green arrows show the chimeric pathway, while the purple arrows show the plant pathway.

Large-scale fermentations are made possible by the employment of microorganisms, and the lack of competing pathways improves future procedures [92]. The yeast *Saccharomyces cerevisiae* and the bacteria *Escherichia coli* are the most often utilized microbes for this purpose. In general, genetically modified microorganisms have several benefits, including the ability to grow in low-cost substrates, simplicity in manipulation, and quick production cycles that enable greater and faster output. *Saccharomyces cerevisiae* and *Escherichia coli* have excellent characterizations and are simple to manipulate, cultivate, and scale up. Additionally, *Saccharomyces cerevisiae* has internal compartments like plants and can undergo post-translational alterations like glycosylation. Furthermore, it is classified as food-grade, which permits its usage in pharmaceuticals and human nutrition [92,93]. A simplified route of RA synthesis using both plant and chimeric pathways in *Saccharomyces cerevisiae* is presented in Figure 3.

When it comes to RA synthesis, finding the best enzyme combination is the first step in achieving the highest conversion rate of intermediates to the desired final product [23]. Several candidates for the genes *CYP98A14*, *RAS*, and *TAT* with coding DNA sequences are available [33,48,94,95] as a result of studies conducted over the past ten years on the major enzymes involved in the production of RA across numerous plant species of the Lamiaceae family [23]. In this context, the BAHD family member—RA synthase LaAT1—from lavender (*Lavandula angustifolia*) was chosen for yeast expression and cloned separately on a vector containing the Arabidopsis gene At4CL5 (which encodes 4-coumarate/CoA ligase and is active on benzoates and hydroxycinnamates). When suitable combinations of donors and acceptor molecules were added, the diverse strains of *Saccharomyces cerevisiae* obtained for the co-expression of *At4CL5* with the different BAHDs successfully produced a wide array of useful hydroxycinnamate and benzoate conjugates. Specifically, for the first time, the synthesis of glycerol hydroxycinnamate esters, quinate hydroxycinnamate esters like chlorogenic acid, as well as RA and its derivatives was demonstrated in yeast [90]. Furthermore, plasmids were used to express the key gene for the synthesis of the RA precursor in *Saccharomyces cerevisiae* to produce salvianolic acid B. RA was produced using a cell-free biosynthetic method in addition to de novo synthesis from basic carbon sources. An ATP and CoA double-regenerating system further raised the titer of RA to 320.04 mg L^−1^ by adding cofactors and substrates [96]. In the study of Babaei et al. [23], *Saccharomyces cerevisiae* was used for constructing a de novo biosynthetic pathway of RA utilizing glucose as a carbon source, and the ultimate titer of RA was 5.93 mg L^−1^. According to the authors, native cytochrome P450 reductase of *Saccharomyces cerevisiae* (*NCP1* gene, accession code P16603) has 31% identity to the protein sequence of *CPR* from two RA producer cells, *Salvia officinalis* and *Plectranthus scutellarioides*, which makes *Saccharomyces cerevisiae* a potential host for RA production. On the other hand, an artificial L-tyrosine-containing RA biosynthetic pathway was created using 4-hydroxyphenylpyruvate as the only node in *Escherichia coli* in order to simplify the biosynthesis pathway [97]. An RA-producing strain of *Saccharomyces cerevisiae* was constructed in the study of Zhou et al. [98] by introducing *OD-LDH^Y52A^* (optimized d-lactate dehydrogenase gene mutant), *OMoRAS*, and *OPc4CL2* into a hyper-producer of caffeic acid. In brief, the final strain YRA113-15B showed a 63-fold improvement compared to the initial strain and produced 208 mg L^−1^ RA in a shake-flask culture.

## 5. Future Perspectives

The latest approach to the synthesis of RA involves utilizing biotechnological techniques, metabolic engineering, and elicitors. In our opinion, the efforts of scientists should be focused on the optimization of cell suspension cultures, which enable the repeatable and high production of RA under controlled conditions. Future research should result the development of detailed RA production protocols for various plant species, which will be available to anyone interested in this subject. Such availability will help extend research and applications in many fields, such as agriculture and medicine. By sharing these protocols, we can promote collaboration and innovation that will ultimately lead to the improved, environmentally friendly production of beneficial compounds like RA.

For RA production, it seems reasonable to use disposable single-use bioreactors, for example, in wave-mixed systems. These easy-to-use bioreactors have been successfully used for many species, and the extraction of secondary metabolites is cost-effective and environmentally friendly. Plant metabolic engineering offers a toolbox for overexpressing or silencing genes to control RA biosynthetic pathways in cells. Suppression of competing pathways, such as lignin production, increases the biosynthesis of phenolic precursors. In contrast, the overexpression of key genes in the RA biosynthetic pathway, such as *c4h*, *tat*, and *hppr*, leads to increased RA accumulation in transgenic lines.

By imitating environmental stress, elicitors activate the plant’s defense mechanisms and promote the synthesis of secondary metabolites such as RA, which are naturally produced as stress-protective substances. The use of elicitors such as MeJA, SA, and YE significantly increases the production of RA by activating its biosynthetic pathways. Elicitors also promote the expression of important genes and enzymes involved in RA production. In addition, elicitation is a simple and inexpensive method to increase RA production in controlled biotechnological systems, so elicitors can be used not only within in vitro tissue culture but also in soil and hydroponics. Combining elicitors with hydroponic cultures of medicinal plants is one of the new trends in organic farming, and the controlled conditions of this system allow for regular monitoring of the concentration of the obtained compounds. The cost-effectiveness of RA production using this method is also important, as it is crucial to meet the growing demand across various industrial sectors, including pharmaceuticals and nutraceuticals. Together, these methods constitute a bio-sustainable and innovative strategy for RA production, overcoming issues such as the overexploitation of natural plant resources and the effects of climate change.

## 6. Conclusions

In the plant world, the biosynthesis of RA begins with the aromatic amino acids L-phenylalanine and L-tyrosine, following their additional independent transformation. Among the enzymes involved in RA biosynthesis, RAS can be regarded as the most specific. Based on the BAHD acetyltransferase enzyme, some key differences can be observed among various members of the Lamiaceae and Boraginaceae families. Furthermore, recent progress has been made pointing to the distinct evolution of RASs in species from the abovementioned families. Both in vitro and in vivo studies have demonstrated that RA, a naturally occurring chemical compound, has great therapeutic potential. Additionally, RA is crucial for plant defense mechanisms against both biotic and abiotic stressors, as well as for promoting plant development. Although it is difficult to obtain high concentrations of RA from natural sources, with the application of the appropriate elicitor (MeJA, SA, or YE) on non-differentiated plant cell cultures in vitro, substantial amounts of RA can be obtained. Studies on this subject demonstrate that various elicitors may influence the expression of the key genes of the phenylpropanoid pathway, including *PAL*, *TAT*, *HPPR*, *4CL*, *C4H, RAS*, and *CYP98A14,* leading to the correlated accumulation of RA. Still, it should be considered that a variety of responses can be obtained due to heterogeneity and differences in the elicitor’s way of action. Due to the recent progress in synthetic biology techniques, RA can also be effectively produced via microbial cell factories, with *Saccharomyces cerevisiae* and *Escherichia coli* as potential hosts to produce both RA and its derivates.

Research on RA needs to be extended to different crop species, considering developments in genomics, transcriptomics, and metabolomics technologies. Additionally, bioinformatical analysis must be combined. Furthermore, additional work is necessary to fully understand the receptors and signaling pathways involved. This will likely expand the range of processes and applications of rosmarinic acid synthesis. Taking all this into account, RA can be regarded as a promising subject for future investigation. Future work is expected to focus on expanding our understanding of the regulatory networks of rosmarinic acid from different plant taxa, broadening the application domains, and balancing the interactions between cell and tissue culture, the environment, and other organisms, building on the current foundations of rosmarinic acid research. The study and use of RA should be further encouraged to guarantee the stability and safety of products.

## Figures and Tables

**Table 1:** Brief description of rosmarinic acid accumulation upon elicitation and process that elicitors influence in different plant species.

Plant Species	Elicitor Used	Culture Conditions	Experimental Outcomes	References
*Salvia officinalis* *Salvia verticillata*	50 μM MeJA and 15 μM AgNO_3_	Foliar application of elicitors	MeJA and Ag^+^ influenced the expression of the key genes *PAL*, *TAT*, *HPPR*, *RAS*, and *CYP98A14* in both phenylpropanoid and tyrosine pathways	[67]
*Melissa officinalis*	150 μM MeJA	Aerial plant parts at vegetative development stage were sprayed	RA accumulation was associated with the transcript level of *MoPAL*, *Mo4CL*, and *MoRAS*	[68]
*Prunella vulgaris*	100 μM MeJA	Hairy root culture	RA accumulation linked with transcript expression of *PvPAL*, *PvHPPR*, *PVC4H*, *PvCL1*, *PvCL2*, and *PvCYP98A101*	[69]
*Salvia yangii* *Salvia abrotanoides*	150 μM MeJA	Aerial plant parts at vegetative development stage were sprayed	1.66- and 1.54-fold increase in RA content due to the increased number of RAS, 4CL, and PAL transcripts	[70]
*Satureja khuzistanica*	100 μM MeJA	Cell suspension culture	Elicitor tripled RA production (3.9 g L^−1^)	[71]
*Lepechinia caulescens*	300 μM MeJA	Hairy root culture	The highest concentration of RA was 24 h after elicitor treatment	[72]
*Agastache rugosa*	50 μM MeJA	Cell cultures	Increased transcript levels of *ArPAL*, *Ar4CL*, and *ArC4H*	[73]
*Mentha* × *piperita*	100 μM MeJA and 200 μM jasmonic acid	Cell suspension culture	1.5 times higher RA concentration (117.95 mg g^−1^ DW and 110.12 mg g^−1^ DW) than in elicitor-free culture	[74]
*Salvia miltiorrhiza*	50 μM MeJA	transgenic lines with hairy roots	*SmMYB1* overexpression increased RA accumulation	[75]
*Origanum vulgare*	0.1 mM MeJA, and *Azotobacter chroococcum*, *Azospirillium brasilense*, *Pseudomonas fluorescens* consortium	MeJA solutions were sprayed on aerial parts of the plants	The expression of RAS and C4H genes increased 3.37 and 6.6 times, respectively	[76]
*Salvia nemorosa*	0.5 μM SA	Callus culture	8-fold increase in RA content compared to the control	[77]
*Salvia miltiorrhiza*	0.16 mM SA	Cell cultures	SA up-regulated the expression of *TAT*, *PAL*, and *RAS* and enhanced the RA accumulation	[78]
*Thymus membranaceus*	10 μM SA	In vitro shoot culture	Increased RA and phenolic levels	[79]
*Salvia officinalis* *Salvia virgata*	250 and 500 μM SA	In vitro shoot culture	Up-regulation of *SoPAL* and *SvPAL* caused by elicitor treatments	[80]
*Salvia miltiorrhiza*	SA and H_2_O_2_	Cell cultures	Synergistic effects of applied elicitors positively influenced RA accumulation and PAL activity	[81]
*Thymus vulgaris*	3 mM SA	Drought-induced stress	Increased RA accumulation	[82]
*Melissa officinalis*	100 mg L^−1^ SA	Foliar application of elicitor	The application of SA in combination with chitosan lactate had the strongest impact on RA accumulation	[83]
*Agastache rugosa*	500 mg L^−1^ YE and 30 mg L^−1^ silver nitrate	Cell suspension culture	4.98 mg L^−1^ of RA after YE elicitation with several times higher transcript levels of *HPPR*	[84]
*Melisa officinalis*	0.1% YE	Elicitor applied on 30-days old seedlings	The highest *TAT* gene expression in relation to RA accumulation	[85]

RA—rosmarinic acid; MeJA—methyl jasmonate; SA—salicylic acid; YE—yeast extract.

## Data Availability

No new data were created or analyzed in this study.
